# The impact of health insurance enrollment on health outcomes in Kenya

**DOI:** 10.1186/s13561-023-00454-7

**Published:** 2023-08-16

**Authors:** Mercy G Mugo

**Affiliations:** Department of Economics & Development Studies, University of Nairobi, P.O. Box 30197, 00100 Nairobi, Kenya

**Keywords:** Health insurance, Health outcomes, Instrumental variable estimation, Kenya

## Abstract

**Background:**

The achievement of the global agenda on universal health coverage (UHC) is pivotal in ensuring healthy lives and promoting the well-being of all. However, achieving healthy lives and wellbeing of all has been hampered by the challenge of health care financing. As such, healthcare financing, through health insurance is gaining popularity in developing countries such as Kenya, in their pursuit to achieve universal health coverage. The primary purpose of health insurance and delivery is to improve health. However, there is a paucity of evidence on the effectiveness of health insurance in improving the health outcomes and health status of the Kenyan population. Therefore, this study aimed to analyze the impact of health insurance on health outcomes in Kenya.

**Methods:**

The study utilized the most recent nationally representative Kenya Integrated Household Budget Survey (KIHBS) 2015/16 dataset in order to analyze the impact of health insurance on health outcomes. The instrumental variable 2-stage least squares (IV 2SLS) and control function approach (CFA) estimation techniques were used to cater for potential endogeneity and heterogeneity biases present in ordinary least squares (OLS) estimators.

**Results:**

Health insurance enrolment leads to a reduction in mortality, thereby improving the health status of the Kenyan population, despite low levels of insurance uptake. However, the insured population experienced higher chronic illnesses and out-of-pocket (OOP) expenditures raising concerns about financial risk protection. The fact that health insurance is linked to chronic illnesses not only reinforces the reverse causality of health insurance and health status, but also that the effects of potential adverse selection strongly drive the strength and direction of this impact.

**Conclusions:**

We conclude that health insurance enrolment reduces mortality and hence has a beneficial impact in promoting health. Health insurance coverage therefore, should be promoted through the restructuring of the National Hospital Insurance Fund (NHIF) fragmented schemes and by consolidating the different insurance schemes to serve different population groups more effectively and equitably. The government should revisit the implementation of a universal social health insurance scheme, as a necessary step towards UHC, while continuing to offer subsidies in the form of health insurance to the marginalized, vulnerable and poor populations.

## Background

The United Nations (UN) sustainable development goal number three’s (SDG3) focus on ensuring healthy lives and promoting well-being for all at all ages, highlights the priority of health in the international development agenda [1]. The World Health Organization’s (WHO) global agenda on Universal Health Coverage (UHC) called for access of all people to quality, comprehensive and effective health services at affordable costs without financial hardship [2, 3]. However, many low- and middle-income countries (LMICs) rely extensively on out-of-pocket (OOP) expenditures to finance health care services with adverse consequences on the use of services and with the risk of financial catastrophic expenditure and impoverishment [4]. Expansion of health insurance coverage is one strategy to attain SDG3 and UHC goals. Economic theory suggests that people purchase health insurance not only to avoid the risk of financial loss, but also as a mechanism for gaining access to healthcare that would otherwise be unaffordable [5–7]. Healthcare financing through health insurance is gaining popularity in developing countries in their pursuit to achieve UHC [8, 9]. Cutler and Zeckhauser [10] posit that the primary purpose of health insurance and its delivery is to improve health. Good health increases productivity, lifetime earnings, and time spent in activities that maximize utility [11, 12]. People care about health insurance because they are concerned about their health [13] and evidence suggests that the effect of being without health insurance can be significant [14, 15]. Although a widely held expectation is that increase in health care finance and expenditure translates into health status improvements, the evidence is scarce and inconclusive [13, 16, 17] particularly for LMICs like Kenya.

Though studies examining the impact of health expenditures on health outcomes mostly document positive associations [18–20], experiences differ by region and health measures. Bein et al. [19] found a positive association between health expenditures and life expectancy while that of neonatal, infant and under five deaths was negative, in eight East African countries. Akinci et al. [21] found that increases in total healthcare expenditures reduce infant, under-five, and maternal mortality rates for Middle Eastern and Northern African countries. However, Anyanwu and Erhijakpor [22] documented region-dependent relationship between health expenditures and infant and under-five mortality whereby it was positive for Sub-Saharan African (SSA) countries and negative for Northern Africa. In countries with good governance, increased health care spending was associated with improvements in infant and under-five child mortality [23].

Most studies focus on the impact of health insurance on health care utilization and OOP, with evidence suggesting that utilization is higher among the insured [4, 9, 14, 24–29] and benefiting those of low socio-economic status significantly more, as was the case for Ghana [30]. However, in their systematic review, Acharya et al. [31] found no strong evidence of impact of insurance on utilization, protection from financial risk and health status in LMICs and caution that increased utilization may not always indicate welfare improvements. Rather, they note that a few schemes afford protection from high OOP, but the impact is weaker on the poor. In South Africa, though private health insurance increased health service utilization, it did not result in lower OOP payments for scheme members compared to nonmembers, raising questions on the effectiveness of insurance in protecting against financial risks and catastrophic expenditures [32].

Proponents of the health insurance strategy argue that health insurance certainly increases the quantity of health care consumed, and many medical interventions have proven to be greatly beneficial [16] especially to some population groups [17]. However, arguments abound that more medical care is not always better and could actually be worse, leaving the question of health benefits of insurance uncertain. Furthermore, there are also other initiatives such as EPI (Expanded Programs on Immunization) or direct investments for medical care and health systems improvements that improve health besides health insurance, evidently raising questions about its effectiveness in improving population health. Aron-Dine et al. [33] note that over three decades later, the Rand Health Insurance Experiment (HIE) results [17, 34] are still widely held to be the “gold standard” of evidence for predicting the likely impact of health insurance reforms on medical spending, health care utilization and health outcomes. Rand [17] states that cost sharing in general had no adverse effects on participant health outcomes though there were exceptions. The poor and sickest patients achieved better outcomes with blood pressure control, vision control and dental care in more generous plans [13, 34]. There was a 10% reduction in mortality for those with hypertension [17]. The study suggested that cost sharing should be minimal or nonexistent for the poor, especially those with chronic disease [7, 17]. Currie and Gruber [35, 36] found that extending public insurance to pregnant women and children was associated with significant decreases in infant and child mortality and with increase in the use of preventive care among children in United States of America (USA).

Culyer and Newhouse [13] cite studies which show that the introduction of universal health coverage in Canada was associated with a decrease in the infant mortality rate. Dor et al. [37] show that private health insurance significantly improved health of older adults (45–64 years) in USA. Levy and Meltzer [16] concluded that their review showed some evidence to support the view that health insurance significantly improves health for specific health measures (e.g., control of hypertension, vision and dental health) or subpopulations (e.g., children, pregnant women, older working age adults). In addition, Dor et al. [37] concluded that there appears to be a general agreement that insurance effects are positive and persist under a variety of disease conditions classifications (chronic, non-chronic, symptomatic, hypertension). The general observation from these studies is that the impact of health insurance on health status cannot be generalized to different settings and population groups, much less in LMICs.

Very few studies examine the impact of health insurance on health outcomes in LMICs [31], with mixed findings. Wang et al. [28] used EuroQol-5 Dimension (EQ-5D) to measure health and report that insurance had positive effects on the health status for all insured and for the poor in China while Mensah et al. [38] showed lower levels of infant deaths in Ghana, though not statistically significant. Stone et al. [14] analyzed all inpatients discharged from a Kenyan teaching and referral hospital to establish the relationship between in-hospital death (mortality) and health insurance status. They found that 21.3% of 956 patients discharged had insurance (mostly National Hospital Insurance Fund (NHIF)) and that the proportion that died was more than twice as high among the uninsured (24.7%) compared to the insured (11.4%). Their findings suggest that insurance coverage was associated with decreased in-hospital mortality and called for expansion of insurance coverage. Haushofer et al. [39] used a randomized controlled trial in Kenya to assess the impact of providing health insurance on self-reported stress and stress hormone cortisol and demonstrated that health insurance reduced both health indicators among informal workers. Insurance was also found to improve sleep and had larger effects on the poor and vulnerable [39]. The foregoing reveals that that there is a paucity of studies that explicitly examine the impact of health insurance on health outcomes at the population level and particularly in SSA. Despite widespread efforts to expand health insurance in developing countries, there is scant evidence as to whether doing so actually improves people’s health [28].

Kenya has made a commitment to achieve UHC. Indeed, one of “*The Big Four*” agenda priority policy objectives of the Kenyan Government is to focus on initiatives that guarantee universal health care to all Kenyans by 2022, through health insurance [40]. The Government of Kenya’s (GOK) decision to implement its UHC initiatives through NHIF was a major achievement towards UHC [41]. Another milestone towards achievement of UHC in Kenya is the 2021 NHIF amendment bill, assented into law in January 2022 [42]. This is part of the GOK’s plan for achieving UHC, based on need rather than, the ability to pay. The bill stipulates that, all Kenyans must be registered under NHIF, in the new Universal Health Coverage (UHC) program, with the government committing to cover the poorer households. The success of UHC will largely depend on a reformed NHIF that improves efficiency, transparency, accountability and service quality [43, 44]. Although enrollment in health insurance in Kenya is low, it has been expanding in recent years [45–48]. Evidence on the question of whether health insurance improves health outcomes at the population level is important in relation to policy questions on the value of initiatives to expand coverage. This evidence is limited for Kenya. Furthermore, the paucity of impact assessment studies, differences in impacts given study populations, settings and disease conditions studied, constitutes knowledge gaps for Kenya as it strives towards UHC.

To address this knowledge gap, we utilized the most comprehensive and nationally representative Kenya Integrated Household Budget Survey (KIHBS) 2015/16 dataset to analyze the impact of health insurance on health outcomes in Kenya. To contextualize the reporting of the results on the impact, we analyzed the characteristics of the insured and uninsured populations in Kenya. We used the instrumental variables (IV) and control function estimation approaches to address potential endogeneity due to omitted variables, selection bias as well as heterogeneity. Tests of significance of differences in means and proportions across health status by insurance status and other control variables were performed using pairwise comparison and *t-test* statistics.

### Kenya’s healthcare financing through insurance

Kenya has a mixed health financing system that relies on several sources of financing. These include revenues collected by the government through taxes and donor funding, the NHIF through member contributions, private health insurance, and out-of-pocket spending by citizens at points of care [8, 49]. Kenya’s health system financing is predominantly tax-funded, through budgetary allocations to the Ministry of Health (MoH) [49]. The government caters for about a third of financing with the rest being private and mainly OOP payments [48]. Existing studies indicate high OOP spending in Kenya [50, 51], with attendant catastrophic and impoverishing expenditures [52–54].

Kenya has not yet implemented a universal social health insurance scheme to date. The President declined to assent to a national social health insurance fund (NSHIF) Bill introduced in 2004 [51]. The health insurance landscape in Kenya consists of public, private, and community-based insurance schemes. The NHIF, established in 1966, is the government mandatory insurance scheme for those in the formal employment sector (private and public) and voluntary for those in the informal sector [55]. NHIF dominates the health insurance landscape due to its mandatory scheme for all formal employees and the low premiums, especially for the informal sector enrollees. Membership in private health insurance offered by general insurance firms as one of their products and largely driven by profit motives rather than health promotion is voluntary. It is usually very expensive and only affordable to the middle- and high-income population groups [51]. Other forms of private insurance include community-based health insurance schemes whose growth has been minimal [51]. Health insurance coverage in Kenya has remained below 20% of the population to date [45–48, 55, 56]. Munge et al. [48] revealed that the majority (88%) of Kenya’s insured population is covered by the NHIF, while the rest are covered by private health insurance. The growth in NHIF enrollment in the last ten years is attributed to reforms largely aimed at increasing coverage among those employed in the informal sector [8]. Private insurance coverage is low (4% of the population) and mainly concentrated in urban areas and among the middle- and high-income groups [51]. In Kenya, it is common for individuals to have more than one insurance scheme. As such, there is no mutual exclusivity in enrollment into any scheme.

The NHIF operates three main schemes namely; the civil service scheme (CSS), the national scheme (*SupaCover*), and health insurance subsidy for the poor (HISP). Each scheme offers different benefit packages with considerable variation between inpatient and outpatient care [8, 57, 58]. NHIF also implements the free maternity, older persons and persons living with severe disabilities, and secondary school students’ insurance programs in order to enhance social protection and inclusivity [58].

Informal sector workers enroll into NHIF by paying a fixed annual premium of KES6,000 (USD 59.9)^1^ for *SupaCover*. Coverage rates are low and attrition is high among this group [8, 48], posing universal coverage challenges. Formal sector employees pay a graduated monthly rate based on salary scale, ranging from Kenya Shillings (KES)150 (USD 1.5) to KES1,700 (USD 17) [55, 58]. Co-payments vary across public (category A), private faith-based (category B) and high cost private (category C) hospitals. NHIF outpatient benefits are based on a positive list of services and payments are made through capitation based on the number of persons registered at a particular facility [59]. The capitation rate ranges between KES 1000 (USD 10) and KES 1400 (USD 14) per beneficiary. Inpatient payment rates for category A and B facilities are 100%, and up to a maximum of KES4,000 (USD 39.9) per day in category C [58]. The CSS benefit package, launched in 2012 was negotiated by the government and NHIF and is delivered through a capitation model at a rate of KES1,500 (USD 15) per annum for public facilities, and KES 2,850 (USD 28.4) for private facilities. Benefits and health facility access vary for civil servant job-group cadres who enjoy a better and wider benefit package than *SupaCover* [8, 57]. The vulnerable and special groups are exempted from premium payments and enjoy the *SuperCover* benefit package. Though heterogenous in scheme types, premiums and benefit entitlements, Kenya’s health insurance model resembles that of many LMICs pursuing healthcare financing reforms geared towards UHC [30, 31].

## Methods

### Data source

We use data from the Kenya Integrated Household Budget Survey (KIHBS) 2015-16. The survey, conducted by Kenya National Bureau of Statistics (KNBS) in 2015-16, is nationally representative and comprehensive in coverage. The survey details are described in KNBS [60]. Through multistage sampling a total of 2,400 clusters (urban = 988; rural = 1,412) were sampled from national sample survey and evaluation programme-five (NASSEP V) sampling frame. The second stage involved selection of 16 households from each of the clusters and the third stage sub-sampling of 10 households. The sample size was determined independently for each of the 47 counties, resulting in a national sample of 24,000 households and 92,768 individuals. We extracted data by merging four sets of questionnaires and associated modules to obtain our variables of interest. This resulted in 21,773 individuals. The IV 2SLS and control function estimation is based on 2,789 individuals due to listwise deletion in Stata.

### Outcome measures and independent variables

We used mortality and chronic condition experience as measures of health outcomes. Both are binary variables indicating presence of death in the household in the last 24 months prior to the survey and presence of chronic illness in the last four weeks respectively. Chronic illness variable was reconstructed by using information on the type of illness or injury suffered and recoded into a binary variable (chronic = 1 and non-chronic illnesses = 0) based on Bernell and Howard [61]. Health insurance status is a binary variable where 1 = insured and 0, otherwise. It comprised of enrolment in any health insurance scheme. The choice of explanatory variables was guided by theoretical and empirical studies. Literature reviewed suggested that demand for health insurance and subsequent impact on health outcomes differs by socio-economic and demographic characteristics [7, 11, 13, 17, 34, 37, 39]. The variables included age, age groups [9, 32, 37, 56] gender [32, 51], marital status [9, 32, 37, 51, 56], religion [62], education [9, 11, 37, 51, 62], media exposure [56], source of energy [9], drinking water [9], residence [9, 56, 62], household size [9], employment status [9, 32, 51, 56, 62], income quintiles and out-of-pocket expenditures [4, 32, 37, 51, 56, 62]. These were used as control variables. In addition to proportion of individuals with insurance within a cluster, we included community level characteristics such as distances to basic services, as additional instruments [63–65].

### Identification Strategy

The gold standard for assessing causal effects is randomized control trial (RCT) such as in the classic Rand HIE [17, 66], but this is rare particularly in LMICs settings. In their review of the impact of health insurance on health, Levy and Meltzer [16] state that many of the studies claiming to show a causal effect of health insurance on health suffer methodological inadequacies because the observed correlation between insurance and good health may be driven by other, unobservable factors. Enrolment into any health insurance scheme may be based on specific population needs that may self-select into insurance schemes and thereby possess different characteristics and outcomes from the non-enrolled population, leading to selection bias. Selection bias arises when a factor affecting participation is associated with the outcome variable. For instance, the sickly may enrol due to a known need for health care services while the healthy may opt out, in both instances self-selecting whether to participate or not. It is plausible to assume that there is a bi-directional causality between health status and health insurance uptake. An individual’s health insurance status is potentially determined by some similar factors that determine health status. As such health outcomes for the insured and uninsured individuals, on one hand, may result from health insurance status or differences between individuals by insurance status both observable and unobservable. On the other hand, health status can also directly affect insurance uptake. This bi-directional causality raises estimation problems [16]. In Kenya, enrolment into insurance is not a random process as people chose whether or not to enrol. Some are enrolled through mandatory requirements in the NHIF whereby they are selected by virtue of being in formal employment while those employed in the informal sector voluntarily take up NHIF. In addition, those with NHIF may also voluntarily purchase private health insurance.

Instrumental variables estimation is one of the empirical strategies that exploit the exogenous variation created by natural experiments, such that, health insurance varies in a way that is unrelated to unobservable characteristics that also determine health, allowing isolation of the causal effect of insurance on health. Although the instrument introduces an element of randomness into treatment assignment, approximating the effect of an experiment, it is extremely difficult but possible to identify relevant, valid and strong instruments [4, 66–68]. Furthermore, the control function approach corrects for potential bias due to non-linear interactions of unobservable variables with the observed regressor [63, 64, 69]. Previous studies [13, 30, 70] suggest that the proportion of the population at a cluster level with insurance, can be treated as exogenous, and therefore a potential instrument for health insurance.

### Analytical models

We used instrumental variable (IV) estimation and control function approach (CFA) to establish the causal effects of health insurance on health status. Potential endogeneity between health status and health insurance may arise from errors-in-variables, omitted variables and simultaneous causality [71]. If the error terms of the health (mortality, chronic illness) production equation and the linear projection of the endogenous variable (insurance status) are correlated it means, there is some unobserved trait that makes people who purchase insurance more or less likely to be healthy in a future period, thereby biasing the coefficients. Dor et al. [37] gives an example: if insurance is positively correlated with an unobserved trait, e.g. ‘‘awareness’’ which leads a person to take better care of their health, the error terms would be positively correlated, biasing the coefficient of insurance upwards. The opposite would hold if the unobserved trait causes a person to neglect their health. The final direction of the simultaneity bias cannot be ascertained a priori. Mwabu [64] observes that endogeneity and heterogeneity bias, compromise the validity of OLS estimators, requiring approaches that exogenize endogenous regressors such as health insurance status. The intent of the IV approach is to exogenize the endogenous regressors using valid, relevant and strong instruments [71]. The instrumental variable approach requires that the instruments are tested for relevance, strength and validity. In addition, in order to use the approach, it is necessary to ascertain that the endogenous variable is indeed endogenous and that the coefficients of the control variables are zero, signifying absence of heteroskedastic errors. The estimation technique uses a single equation approach using two-stage least squares (2SLS) estimators [64, 71, 72].

The IV method is one of the most powerful tools in econometrics, because it allows consistent parameter estimation in the presence of correlation between explanatory variables and disturbances i.e. endogeneity [73]. The method also identifies causal or treatment effects and it essentially assumes that some components of non-experimental data are random [65]. Instruments are variables thought to have no direct association with the outcome (health outcomes) [74] and are powerful predictors of treatment (health insurance) [72]. Though finding relevant, valid and strong instruments is difficult, techniques for testing for these qualities exist [75–77] as discussed under diagnostic tests below. Following the literature [13, 16, 30, 35, 37] we use the proportion of individuals with insurance within a cluster excluding the individual as an instrument for health insurance. The argument is that the individual does not affect this proportion and is therefore treated as exogenous.

Equations 1–3 summarise the empirical specification for IV 2SLS and CFA based on Mwabu [64] and Baye and Fambon [78] with adaptation from Bascle [71].

1 $$Yi\;=\;\beta_1X_1\;+\;\beta_2M_{li}+_{...}+\;\beta_{i+r}M_{ri}\;+\;\mu_i$$

2 $$X_{i}=\pi_{1}V_{1i}+{...}+\pi_{m}V_{mi}+\pi_{m+1}M_{1i}+{...}+\pi_{m+r}M_{mi}+\upsilon_{i}$$

Where: *Y*_*i*_ represents health outcome (mortality, chronic illness); *X*_*i*_ is endogenous determinant of health status, i.e. health insurance; *M*_*1i….*_*M*_*ri*_ are each of the *r* exogenous covariates or control variables, such as socio-demographics; *V*_*i*_ are a vector of instruments that affect the endogenous determinant of health status (health insurance) but have no direct influence on health status; *β* and *π* are the parameters to be estimated; *u*_*i*,_ and *υ*_*i*_ are the disturbance terms. Equation 1, represents the health production technology and is the structural equation of interest. Unobserved complementarity between health insurance and other inputs omitted from this equation, may result in reverse causality. This is eliminated through the use of instrumental variables that do not belong to Eq. 1 but are strongly correlated with the endogenous determinant of health production; health insurance. Equation 2 is a linear projection of health insurance on all the exogenous variables, *M*_*1i….*_*M*_*ri*_ and *V*_*i*_. Equation 2 is a reduced form linear probability model of health insurance (the endogenous) input into health production. The predicted values of health insurance are used to compute residuals that enter Eq. 3, which is Eq. 1 augmented into a control function. To correct for potential endogeneity and non-linear interactions of unobservable variables with the observed regressor specified in Eq. 1, the equation is extended as indicated in Eq. 3.

3 $$Y_{i}+\beta_{1}X+\beta_{2}M+\delta\,R+\gamma\left(R\,\mathrm{x}\,X\right)+\mu_{i}$$

*Y*_*i*_ is as previously defined, *X* is previous *X*_*i*,_*M* is previous *M*_*1i, ….*_*M*_*ri*_. *R* is the residual of the endogenous input (i.e. observed value of X minus its fitted value), *(R* x *X )* is the interaction of the residual with an endogenous input. *δ* and *γ* are parameters to be estimated. The terms *R* and *(R* x *X)* in Eq. 3 are the control function variables. They control for the effects of unobservable factors that would contaminate the OLS estimates of the structural parameters of health status measures. *R* serves as a control for unobserved variables correlated with *X*, thus allowing *X* to be treated as though they were exogenous during estimation. *(R* x *X)* controls for effects of neglected non-linear interactions of the unobservable variables with the health status measures. Equation 3 is the correct specification in the absence of a priori information on the econometric problems present [64]. This equation was estimated using the maximum likelihood estimation (MLE) procedure in Stata/SE 14.1 statistical software.

### Diagnostic tests

The Durbin and Wu-Hausman test statistics determine whether the endogenous regressors in the model are in fact exogenous [75, 76, 79, 80]. If the test statistic is significant, then the variables being tested must be treated as endogenous. If the endogenous regressors are in fact exogenous, then the OLS estimator is more efficient, and depending on the strength of the instruments and other factors, the sacrifice in efficiency by using an instrumental variables estimator can be significant. Thus, unless an instrumental variable estimator is really needed, OLS should be used instead. Various statistics are used to measure the relevance and strength of the excluded exogenous variables depending on the number of endogenous regressors. The partial R^2^ and the first-stage F-statistic are reported in case of one endogenous regressor whereas Shea’s partial R^2^ and adjusted partial R^2^ are reported in case of two or more endogenous regressors. Higher values of R^2^ may indicate stronger instruments, though not always the case [74, 77, 79–82].

The partial R^2^ statistic measures the correlation between the endogenous variable and the additional instruments after partialling out the effect of the other exogenous variables in the equation, that may cause higher R^2^. Bound et al. [74] and others have promoted using the partial R^2^ statistic. The minimum eigenvalue statistic is a further test of weak instruments based on Cragg and Donald [81]. If the model contains one endogenous variable the minimum eigenvalue statistic and the F-statistic are identical. The Stock and Yogo [82] minimum eigenvalue statistic tests the hypothesis that the set of instruments are weak. If the test statistic exceeds the critical value, the instruments are deemed strong. The critical values are based on the largest relative bias of the 2SLS estimator tolerable or the largest rejection rate of a nominal 5% Wald test tolerable, and these are provided in Stata output [79]. The F-statistic tests for the joint significance of the coefficients on the additional instruments. If the p-value shows that the F statistic is not significant, then the additional instruments have no significant explanatory power for the endogenous variable after controlling for the effect of the exogenous covariates. Stock et al. [77] suggest that the F statistic should exceed 10 for inference, based on the 2SLS estimator, to be reliable when there is one endogenous regressor. Sargan’s and Basmann’s *chi*^*2*^ statistic test overidentifying restrictions [75, 76]. In addition to the requirement that instrumental variables be correlated with the endogenous regressors, the instruments must also be uncorrelated with the structural error term, thereby assuming that the errors are independent and identically distributed normal. Tests of overidentifying restrictions test two different things simultaneously. One is whether the instruments are uncorrelated with the error term, indicating if the errors are homoscedastic or heteroskedastic. The other, is that the equation is misspecified and that one or more of the excluded exogenous variables should in fact be included in the structural equation. Thus, a significant test statistic could represent either an invalid instrument or an incorrectly specified structural equation. Murray [75, 76] suggests the use of these tests, among other strategies, for supporting instrument validity. Another strategy is to rule out links between instruments and error term based on theoretical reasoning and evidence of non-correlation. Thirdly, use alternative instruments and compare the results i.e. do all the instruments tell the same story? Fourthly, use and check intuition including appealing to economic theory to establish validity. The F-test indicates whether the coefficients on the control variables are zero. If the p-value is not significant, then the hypothesis that the coefficients are zero is supported, signifying that the errors are homoskedastic (i.e. no heterogeneity).

## Results

### Sample description

Table 1 presents descriptive statistics for the sample. Among the 21,773 individuals, 18% were covered by any health insurance. For the majority (94%), NHIF was the first source of health insurance while other sources included contributory private insurance (3.3%), employer contributory (1.2%), and private non-contributory (0.6%). This is an indication of limited levels (4%) of private insurance uptake in Kenya, with negligible contribution from both contributory and non-contributory employer schemes and underscores the prominence of the state-run health insurance scheme. It is worth noting that 38.3% of those with health insurance were employed in the formal sector while about 22% were employed in the informal sector (Table 2). The rest were not employed and therefore presumed to be dependents. The instrument for health insurance, proportion of individuals with insurance in a cluster excluding the individual was 12%. About 23% of individuals experienced OOP expenditures, with mean OOP expenditures of KES15,619 (USD155.8). Mortality was 3.8%, with female-headed households experiencing higher mortality (4.6%) compared to male-headed households (2.6%). Of those that experienced mortality, the majority were not covered by any form of health insurance, suggesting some role for health insurance in mitigating mortality. Similarly, those that experienced mortality incurred OOP expenditures averaging KES12,001(USD119.7) compared to KES14,805 (USD147.7) of those that did not. This is an indication that ability to finance health care through OOP may potentially mitigate mortality through healthcare access. About 24% of individuals had an illness or injury in the last four weeks preceding the survey out of whom 4% suffered chronic condition. Of those who had an illness or injury and suffered chronic condition, 55 and 62.4% respectively were females.

**Table 1:** **Sample description**

***VARIABLES***	***N (% /Mean)***
Mortality	831 (3.82)
Illness experience	5,244 (24.10)
Chronic illness experience	866 (3.98)
Health Insurance status	3,920 (18.02)
Out of pocket expenditures in KES	4,948 (15,619.8)
Household incurred OOP	4,948 (22.73)
Proportion with health insurance per cluster	21,773 (12.00)
Age in years	21,757 (27.19)
Age of household head in years	8,221 (44.01)
Age groups	
Early childhood	2,970 (13.65)
Late childhood	3,516 (16.16)
Adolescence & youth	5,025 (23.10)
Adults	8,162 (37.51)
Elderly	2,084 (9.58)
Male gender	11,153 (51.22)
Male gender of household head	5,282 (64.19)
Marital status	
Married	7,462 (44.53)
Special single^a^	2,446 (14.60)
Single	6,849 (40.87)
Average household size	21,773 (4.26)
Education	
None	7,938 (47.32)
Primary	4,664 (27.80)
Secondary and higher	4,173 (24.88)
Employment status	
Formal	4,112 (18.89)
Informal	7,011 (32.20)
Not working	10,650 (48.91)
Mean income by quintile in KES	
Poorest	4,355 (2,060.07)
Poor-middle	4,355 (3,456.84)
Middle	4,354 (4,990.32)
Middle-rich	4,355 (7,377.54)
Richest	4,355 (15,242.26)
Religion	
Christian	18,236 (83.76)
Muslim	2,876 (13.21)
Hindu and others	661 (3.04)
Rural residence	13,092 (60.13)
Media exposure	5,369 (24.71)
Main source of energy for cooking	
wood-based e.g. firewood, charcoal	19,789 (91.12)
Electricity	129 (0.59)
LPG gas	1,799 (8.28)
Sanitation- Type of toilet facility	
Flush or pour flush	2,501 (11.51)
Pit latrine	16,043 (73.83)
No toilet facility	2,997 (13.79)
Others	189 (0.87)
Drinking water access type	
Piped or bottled water	9,791 (45.05)
Dug well / spring water	6,082 (27.98)
Rain and surface water	4,785 (22.02)
Vendors water	826 (3.80)
Others	250 (1.15)
Dwelling type of walls	
Natural walls e.g. mud, grass	6,999 (32.20)
Rudimentary walls e.g. bamboo, cardboard	4,219 (19.41)
Finished walls e.g. stone, cement	10,210 (46.98)
Others	306 (1.41)
Distance to water source in Kilometers	19,852 (0.79)
Distance to water source-time in Minutes	19,885 (22.12)
Distance to nearest tar/asphalt road in Kilometers	21,415 (21.75)
Distance to nearest bus/matatu stage in Kilometers	21,713 (4.19)
Distance to nearest market in Kilometers	21,704 (8.24)
Distance to nearest public primary school in Kilometers	21,599 (1.26)
Distance to nearest public secondary school in Kilometers	21,619 (4.56)
Distance to nearest health facility in Kilometers	21,725 (2.02)

^a^Defined as previously in a union (separated, divorced, widow/widower)

**Table 2:** Comparisons among Insured and Uninsured Populations

	Insured (N=3,920)	Uninsured (N=17,883)	Difference (Insured – Uninsured)
	N (% /Mean)	N (% /Mean)	
Mortality	(84) 2.14	(789) 4.19	-2.05***
Illness experience	(962) 24.55	(4,281) 24.01	0.54
Chronic illness experience	(185) 04.72	(679) 3.81	0.91***
Out of pocket expenditures in KES	(984) 29,119.95	(3,963) 12,270.02	16,849.93***
Household incurred OOP	(984) 25.01	(3,962) 22.22	2.87***
Proportion with health insurance per cluster	(1,215) 31.00	(1,248) 7.00	24.00***
Age in years	(3,920) 29.22	(17,818) 26.75	2.47***
Age of household head in years	(1,858) 41.5	(6,355) 44.75	-3.24***
Age groups			
Early childhood	(445) 11.35	(2,523) 14.16	-2.81***
Late childhood	(508) 12.95	(3,004) 16.86	-3.90***
Adolescence & youth	(698) 17.81	(4,321) 24.25	-6.44***
Adults	(1,999) 50.99	(6,156) 34.55	16.44***
Elderly	(270) 6.89	(1,812) 10.17	-3.29***
Male gender	(2,163) 55.18	(8,977) 50.34	4.84***
Male gender of household head	(1,342) 72.23	(3,935) 61.84	10.38***
Marital status			
Married	(1,879) 58.79	(5,573) 41.15	17.64***
Special single	(248) 7.75	(2,198) 16.23	-8.47***
Single	(1,069) 33.45	(5,772) 42.62	-9.17***
Household size	(3,920) 3.64	(1,7833) 4.40	-0.76***
Education			
None	(1,000) 28.57	(6,931) 52.26	-23.69***
Primary	(789) 22.54	(3,871) 29.19	-6.65***
Secondary and higher	(1,711) 48.88	(2,459) 18.54	30.34***
Employment status			
Formal	(1,522) 38.82	(2,588) 14.51	24.31***
Informal	(850) 21.68	(6,158) 34.53	-12.84***
Not working	(1,548) 39.48	(9,086) 50.95	-11.46***
Income quintiles			
Poorest	(193) 4.92	(4,159) 23.32	-18.39***
Poor-middle	(375) 9.57	(3,979) 22.31	-12.74***
Middle	(646) 16.47	(3,706) 20.78	-4.30***
Middle-rich	(1,017) 25.94	(3,331) 18.68	7.26***
Richest	(1,689) 43.09	(2,657) 14.90	28.18***
Religion			
Christian	(3,706) 94.54	(14,512) 81.38	13.15***
Muslim	(156) 3.97	(2,718) 15.24	-11.27***
Hindu and others	(58) 1.49	(603) 3.38	-1.89***
Rural residence	(1,724) 43.98	(11,361) 63.71	-19.73***
Media exposure	(2,174) 55.58	(3,190) 17.91	37.67***
Main source of energy for cooking			
Wood-based e.g. firewood, charcoal	(2,906) 74.42	(16,874) 94.78	-20.36***
Electricity	(46) 1.18	(84) 0.47	0.71***
LPG gas	(953) 24.40	(846) 4.75	19.65***
Sanitation- Type of toilet facility			
Flush or pour flush	(1,077) 27.55	(1,423) 7.99	19.56***
Pit latrine	(2,788) 71.32	(13,247) 74.37	-3.05***
No toilet facility	(37) 0.94	(2,959) 16.61	-15.66***
Others	(7) 0.17	(182) 1.02	-0.84***
Drinking water access type			
Piped or bottled water	(2,503) 63.99	(7,282) 40.88	23.12***
Dug well / spring water	(669) 17.11	(5,412) 30.38	-13.28***
Rain and surface water	(548) 14.01	(4,234) 23.77	-9.76***
Vendors water	(156) 3.99	(670) 3.76	0.23
Others	(35) 00.89	(214) 1.20	-0.31*
Dwelling type of walls			
Natural walls e.g. mud, grass	(478) 12.22	(6,518) 36.59	-24.37***
Rudimentary walls e.g. bamboo, cardboard	(459) 11.74	(3,757) 21.09	-9.36***
Finished walls e.g. stone, cement	(2,931) 74.94	(7,273) 40.83	34.11***
Others	(43) 1.09	(262) 1.47	-0.38*
Distances to basic and community level services			
Distance to water source in Kilometers	(3,020) 0.53	(16,823) 0.83	-0.3**
Distance to water source-time in Minutes	(3,026) 12.91	(16,850) 23.77	-10.86***
Distance to nearest tar/asphalt road in Kilometers	(3,905) 6.9	(17,490) 25.07	-18.17***
Distance to nearest bus/matatu stage in Kilometers	(3,912) 1.75	(17,781) 4.73	-2.98***
Distance to nearest market in Kilometers	(3,920) 4.16	(17,764) 9.15	-4.99***
Distance to nearest public primary school in Kilometers	(3,891) 0.96	(17,688) 1.33	-0.37***
Distance to nearest public secondary school in Kilometers	(3,892) 2.01	(17,707) 5.12	-3.11***
Distance to nearest health facility in Kilometers	(3,918) 1.05	(17,787) 2.24	-1.18***

*** *p < 0.01*; **p < 0.05; *p < 0.1

Table 2 compares insured (n = 3920) and uninsured (n = 17,883) individuals using pairwise mean comparison tests of the differences between means for continuous variables and tests on equality of proportions for categorical variables. The uninsured experienced significantly higher mortality which may imply poor access and delays in seeking care, compared to the insured. There were no significant differences in the presence of illness or injury, though the magnitude was higher among the insured. Chronic illness experience was significantly higher among the insured, suggesting adverse selection whereby the healthier population opts out of insurance. This may be driven by perceptions of benefits and costs in relation to health-related risks and insurance premiums, partly explaining the low levels of private health insurance in Kenya. A significantly higher proportion of the insured experienced OOP expenditures. The difference in mean OOP expenditures was positive and significant suggesting that the insured experienced higher OOP expenditures. While this seems contrary to theory and arguments for health insurance which predicts lower OOP expenditures for the insured, several plausible explanations may be advanced for this finding in the Kenyan context. First is the possibility that the insured are more likely to use health care services due to ease of access thereby incurring higher OOP spending in the process, given the insurance package type and extent of co-payment. Secondly, the insured may also need to use more health services in the face of adverse selection and third, is the possibility that the existing health insurance benefit packages are inadequate, requiring the insured to incur OOP expenditures. As expected, a significantly higher proportion of those in formal employments are insured (39% vs. 15%) while a higher proportion of those in the informal sector are uninsured (35% vs. 22%). The proportions of the insured increase with increase in income for the insured, and decrease with increase in income for the uninsured, and the differences are significant. The mean differences between the insured and uninsured are significant for all the socio-demographic characteristics. The insured are older, male, married, educated, Christians, exposed to media, have smaller household sizes and are predominantly urban dwellers. They also have better water and sanitation conditions as well as better access to basic social and community level services.

### Impact of health insurance on health outcomes

Tables 3 and 4 present the OLS, IV 2SLS and CFA results for mortality and chronic illness models respectively. At 5.39 and 5.34, the Durbin *X*^*2*^ score and Wu-Hausman F-statistic were respectively both significant at p < 0.05 indicating that health insurance is endogenous (Table 3). The R^2^ of the first-stage regression was moderate at 0.323 suggesting moderately relevant instruments. Though the adjusted R^2^ is 0.315, partial R^2^ is rather low at 0.171 signifying that the instruments may be weakly relevant. It is worth noting that since we have only one endogenous regressor, the partial R^2^ and the Shea’s partial R^2^ for multiple instrument are identical. That, the first-stage F-statistic (71.09; p-value = 0.000) for testing the joint significance of instruments is greater than 10 indicates that the instruments are strong. At 71.09 (critical value = 20.25) of 5% 2SLS relative bias and 33.84 for a 10% rejection rate of a 2SLS size of nominal 5% Wald test, the minimum eigenvalue statistic further supports the finding that the set of instruments are strong. Sargan’s (6.18) and Basman’s (6.11) test statistics of overidentifying restrictions were both not significant. This suggests that the instruments are valid and that the structural equation is correctly specified. We conclude that the instruments for health insurance status in estimating mortality are weakly relevant, valid and strong and therefore reliable for inference. Furthermore, we establish that health insurance status is endogenous, that the structural equation is correctly specified, and that the IV 2SLS technique is appropriate for estimating the impact of health insurance on health outcomes (mortality).

**Table 3:** OLS, IV 2SLS and Control Function Approach Parameter Estimates of impact of Health Insurance on Health Status (Dependent variable = Mortality)

	OLS	Reduced Form Estimates for Health Insurance Status in IV 2SLS	IV 2SLS	Control function approach
Health Insurance status	0.010 (-0.009-0.030)		-0.048* (-0.103-0.007)	-0.027 (-0.068-0.014)
Out of pocket expenditure in KES	0.00001 (-0.0001-0.0001)	0.0004*** (0.0001-0.0006)	0.00003 (-0.0001-0.0002)	0.00002 (-0.0001-0.0002)
Proportion with health insurance per cluster		0.967*** (0.886-1.047)		
Age in years	-0.0003 (-0.001-0.001)	0.001* (-0.000-0.003)	-0.0002 (-0.0012-0.0008)	-0.0002 (-0.001-0.0008)
Age groups [Reference: early and late childhood]				
Adolescence & youth	0.006 (-0.030-0.043)	0.036 (-0.072-0.145)	-0.008 (-0.079-0.064)	0.004 (-0.036-0.044)
Adults	0.003 (-0.042-0.049)	-0.050 (-0.137-0.038)	-0.004 (-0.062-0.054)	0.004 (-0.046-0.053)
Elderly	0.008 (-0.057-0.073)	-0.012 (-0.072-0.048)	-0.004 (-0.044-0.036)	0.007 (-0.064-0.078)
Male gender	0.002 (-0.013-0.017)	-0.009 (-0.035-0.017)	0.006 (-0.011-0.023)	0.006 (-0.011-0.023)
Marital status [Reference: Married]				
Special single	0.077*** (0.054-0.017)	-0752*** (-0.113- -0.037)	0.078*** (0.052-0.103)	0.079*** (0.054-0.104)
Single	0.004 (-0.017-0.026)	-0.035* (-0.072-0.002)	0.0010 (-0.024-0.025)	0.0014 (-0.023-0.026)
Religion [Reference: Christian]				
Muslim	0.0005 (-0.032-0.033)	-0.050 (-0.108-0.008)	0.004 (-0.034-0.042)	0.005 (-0.033-0.042)
Hindu and others	0.033 (-0.021-0.087)	-0.086* (-0.179-0.007)	0.020 (-0.041-0.081)	0.021 (-0.039-0.083)
Education [Reference: None]				
Primary	-0.13 (-0.032-0.006)	0.044*** (0.012-0.075)	-0.108 (-0.031-0.010)	-0.011 (-0.032-0.009)
Secondary and higher	-0.016 (-0.038-0.006)	0.102*** (0.065-0.140)	-0.007 (-0.033-0.019)	-0.009 (-0.034-0.017)
Media exposure	-0.009-0.029-0.010)	0.109*** (0.075-0.142)	-0.002 (-0.026-0.021)	-0.004 (-0.027-0.018)
Main source of energy for cooking [Reference: Wood-based]				
Electricity	-0.032 (-0.124-0.059)	0.026 (-0.142-0.195)	-0.029-0.141-0.083)	-0.032 (-0.143-0.080)
LPG gas	-0.012-0.0408-0.016)	0.084*** (0.029-0.140)	-0.001 (-0.038-0.036)	-0.003 (-0.039-0.034)
Drinking water access type [Reference: Piped or bottled water]				
Spring water	0.009 (-0.009-0.029)	0.040** (0.009-0.073)	0.006 (-0.014-0.027)	0.006 (-0.014-0.027)
Rain and surface water	0.018 (-0.004-0.029)	0.050*** (0.016-0.085)	0.019 (-0.004-0.042)	0.018 (-0.004-0.041)
Vendors water	0.016-0.024-0.057)	0.019-0.046-0.084)	0.017 (-0.026-0.060)	0.017 (-0.026-0.060)
Others	0.032 (-0.036-0.101)	0.106*-0.005-0.218)	0.019 (-0.055-0.093)	0.018 (-0.056-0.092)
Rural residence	-0.006 (-0.022-0.011)	0.079*** (0.049-0.108)	-0.003 (-0.021-0.016)	-0.003 (-0.021-0.015)
Household size	0.004** (0.000-0.008)	0.012*** (0.005-0.018)	0.004* (-0.000-0.008)	0.004* (-0.000-0.008)
Employment status [Reference: Formal]				
Informal	-0.001 (-0.019-0.018)	-0.111*** (-0.143- -0.079)	-0.010 (-0.032-0.012)	-0.009 (-0.030-0.013)
Not working	-0.009 (-0.032-0.014)	-0.074*** (-0.112- -0.035)	-0.015 (-0.041-0.010)	-0.014 (-0.040-0.011)
Income quintile [Reference: Poorest]				
Poor-middle	0.007 (-0.021-0.037)	0.006 (-0.040-0.053)	0.006 (-0.023-0.038)	0.007 (-0.024-0.038)
Middle	0.002 (-0.026-0.037)	0.062** (0.015-0.108)	0.007 (-0.023-0.038)	0.006 (-0.024-0.038)
Middle-rich	0.009 (-0.019-0.039)	0.075*** (0.028-0.122)	0.010 (-0.022-0.042)	0.0009 (-0.023-0.041)
Richest	0.007 (-0.024-0.040)	0.128*** (0.076-0.180)	-0.014 (-0.022-0.049)	-0.012 (-0.023-0.047)
Distance to water source-time in Minutes		-0.0001 (-0.0005-0.0002)		
Distance to nearest tar/asphalt road in Kilometers		0.0002 (-0.0003-0.0007)		
Distance to nearest bus/matatu stage in Kilometers		0.0007 (-0.001-0.003)		
Distance to nearest market in Kilometers		0.00002-0.001-0.001)		
Distance to nearest public primary school in Kilometres		-0.002 (-0.009-0.006)		
Distance to nearest public secondary school in Kilometers		0.0006 (-0.001-0.002)		
Distance to nearest health facility in Kilometers		-0.003 (-0.007-0.001))		
Health insurance status residuals				0.026* (-0.001-0.053)
Health insurance status X its residual				-0.013 (-0.042-0.016)
Constant	0.025 (-0.032-0.083)	-0.102* (-0.256-0.012)	0.042 (-0.044-0.127)	0.035 (-0.027-0.098)
***Diagnostic tests***				
R2 for the regression	0.023	0.323	0.014	0.025
Durbin			5.39**	
Wu-Hausman			5.34**	
Adjusted R2			0.315	
Partial R2			0.171	
Shea’s Partial R2 (>1 endogenous variable)			0.171	
Adjusted partial R2			0.157	
First Stage F-statistic [joint significance of instruments]			71.09***	
Minimum eigenvalue statistic (Cragg & Donald: similar to First stage F in case of one endogenous variable)			71.09	
Sargan’s tests statistic			6.18	
Basman tests statistic			6.11	
Health insurance status residuals F-test				3.50*
Health insurance status X its residual F-test				0.73
Observations	3,175	2,790	2,790	2,789

95% confidence interval in parentheses. *** *p < 0.01*; ** p < 0.05; * p < 0.1

**Table 4:** OLS, IV 2SLS and Control Function Approach Parameter Estimates of impact of Health Insurance on Health Status (Dependent variable = Chronic Illness)

	OLS	Reduced Form Estimates for Health Insurance Status in IV 2SLS	IV 2SLS	Control function approach
Health Insurance status	0.025 (-0.006-0.057)		0.114*** (0.031-0.198)	0.093*** (0.030-0.156)
Out of pocket expenditure in KES	0.0001 (-0.00008-0.0004)	0.0004*** (0.0001-0.0006	0.0002 (-0.0001-0.0004)	0.0002 (-0.0000-0.0004)
Proportion with health insurance per cluster		0.967*** (0.878-1.047)		
Age in years	0.004*** (0.002-0.005)	0.001* (-0.0002-0.003)	0.003*** (0.001-0.004)	0.003*** (0.002-0.005)
Age groups [Reference: early and late childhood]				
Adolescence & youth	-0.062*** (-0.120- -0.003)	0.036 (-0.072-0.145)	-0.043 (-0.153-0.067)	-0.055* (-0.116-0.005)
Adults	-0.064* (-0.135-0.008)	-0.050 (-0.137-0.037)	-0.098** (-0.187- -0.010)	-0.050 (-0.126-0.025)
Elderly	0.002-0.101-0.106)	-0.012 (-0.072-0.048)	-0.094*** (-0.155- -0.032)	0.044 (-0.066-0.154)
Male gender	-0.033*** (-0.057- -0.008)	-0.009 (-0.035-0.017)	-0.033** (-0.059- -0.006)	-0.033** (-0.058-0.006)
Marital status [Reference: Married]				
Special single	0.029 (-0.007-0.066)	-0.075*** (-0.113-0.037)	0.0429** (0.004-0.082)	0.042** (0.003-0.080)
Single	0.032* (-0.002-0.067)	-0.035* (-0.072-0.002)	0.036* (-0.001-0.073)	0.035* (-0.003-0.073)
Religion [Reference: Christian]				
Muslim	0.015-0.037-0.067)	-0.050* (-0.108-0.008)	0.022 (-0.035-0.080)	0.021 (-0.037-0.079)
Hindu and others	-0. (-0.107-0.063)	-0.086* (-0.179-0.007)	-0.026 (-0.120-0.068)	-0.028 (-0.122-0.066)
Education [Reference: None] Primary	0.005 (-0.025-0.035)	0.043*** (0.012-0.075)	-0.001 (-0.033-0.030)	-0.001 (-0.033-0.030)
Secondary and higher	0.011 (-0.025-0.046)	0.103*** (0.065-0.140)	-0.002 (-0.042-0.037)	-0.0007 (-0.040-0.039)
Media exposure	0.021 (-0.010-0.052)	0.109*** (0.075-0.142)	0.006 (-0.029-0.042)	0.008 (-0.027-0.043)
Main source of energy for cooking [Reference: Wood-based]				
Electricity	-0.041 (-0.186-0.104)	0.026 (-0.143-0.195)	0.030 (-0.142-0.202)	0.033 (-0.138-0.204)
LPG gas	0.019 (-0.026-0.065)	0.084*** (0.029-0.139)	0.018 (-0.038-0.076)	0.020 (-0.037-0.076)
Drinking water access type [Reference: Piped or bottled water]				
Dug well / spring water	-0.015 (-0.046-0.016)	0.041**0.009-0.073)	-0.016 (-0.048-0.016)	-0.016 (-0.047-0.016)
Rain and surface water	-0.002 (-0.036-0.031)	0.051*** (0.016-0.086)	-0.006 (-0.040-0.029)	-0.005 (-0.040-0.029)
Vendors water	-0.060* (-0.124-0.003)	0.019 (-0.047-0.084)	-0.061* (-0.127-0.005)	-0.062* (-0.128-0.004)
Others	0.131** (0.022-0.240)	0.106**-0.005-0.218)	0.147* (0.033-0.261)	0.148* (0.034-0.261)
Rural residence	0.024* (-0.002-0.051)	0.079*** (0.050-0.109)	0.019 (-0.009-0.047)	0.019 (-0.009-0.047)
Household size	0.002 (-0.004-0.008)	0.012*** (0.005-0.018)	0.003 (-0.004-0.009)	0.003 (-0.004-0.009)
Employment status [Reference: Formal]				
Informal	0.0007 (-0.030-0.031)	-0.111*** (-0.143- -0.079)	0.007 (-0.027-0.041)	0.005 (-0.028-0.038)
Not working	0.028 (-0.008-0.065)	-0.074*** (-0.112- -0.035)	0.022 (-0.018-0.062)	0.021 (-0.019-0.060)
Income quintile [Reference: Poorest]				
Poor-middle	0.041* (-0.006-0.086)	0.006 (-0.041-0.053)	0.044* (-0.003-0.091)	0.044* (-0.003-0.091)
Middle	0.032 (-0.013-0.078)	0.062*** (0.016-0.108)	0.033 (-0.014-0.081)	0.033 (-0.013-0.081)
Middle-rich	0.045* (-0.001-0.092)	0.075*** (0.028-0.122)	0.043* (-0.003-0.101)	0.044* (-0.004-0.092)
Richest	0.048* (-0.002-0.099)	0.128*** (0.076-0.179)	0.052* (-0.002-0.105)	0.053* (-0.002-0.107)
Distance to water source-time in Minutes		-0.0001 (-0.0005-0.0002)		
Distance to nearest tar/asphalt road in Kilometers		0.0002 (-0.0003-0.0007)		
Distance to nearest bus/matatu stage in Kilometers		0.0007 (-0.001-0.003)		
Distance to nearest market in Kilometers		-0.00002 (-0.001-0.001)		
Distance to nearest public primary school in Kilometers		-0.002 (-0.009-0.006)		
Distance to nearest public secondary school in Kilometers		0.0006 (-0.001-0.003)		
Distance to nearest health facility in Kilometers		-0.003 (-0.007-0.002)		
Health insurance status residuals				-0.037* (-0.079-0.004)
Health insurance status X its residual				0.017 (-0.028-0.062)
Constant	-0.039 (-0.129-0.052)	-0.102 (-0.233-0.029)	0.005 (-0.126-0.137)	-0.039 (-0.135-0.056)
R2 for the regression	0.075	0.323	0.076	0.084
Durbin			3.91**	
Wu-Hausman			3.87**	
Adjusted R2			0.315	
Partial R2 (one to report)			0.171	
Shea’s Partial R2 (>1 endogenous variable)			0.171	
Adjusted partial R2			0.157	
First Stage F-statistic [joint significance of instruments]			71.09***	
Minimum eigenvalue statistic (Cragg & Donald) similar to First stage F in case of one endogenous variable			71.09	
Sargan’s tests statistic			4.89	
Basman tests statistic			4.84	
Health insurance status residuals F-statistic				2.92*
Health insurance status X its residual F-statistic				0.53
Observations	3,175	2,790	2,790	2,789

95% confidence interval in parentheses. *** *p < 0.01*; ** p < 0.05; * p < 0.1

The Durbin *X*^*2*^ score (3.91: p-value = 0.05) and Wu-Hausman F-statistic (3.87: p-value = 0.05) confirm that health insurance is endogenous in the chronic illness model (Table 4). The R^2^ of the first-stage regression was moderate at 0.323 suggesting moderately relevant instruments. The partial R^2^ is rather low at 0.171 signifying that the instruments may be weakly relevant. However, the first-stage F-statistic (71.09; p-value = 0.000) for testing the joint significance of instruments is greater than 10, an indication that the instruments are strong. At 71.09 (critical value = 20.25) of 5% 2SLS relative bias and 33.84 for a 10% rejection rate of a 2SLS size of nominal 5% Wald test, the minimum eigenvalue statistic further supports the finding that the set of instruments are strong. Sargan’s (4.89) and Basman’s (4.84) test statistics of overidentifying restrictions were both not significant, an indication that the instruments are valid, and that the structural equation is correctly specified. We conclude that the instruments for health insurance in estimation of the impact of health insurance on health outcomes (chronic illness) are weakly relevant, valid and strong. Given that health insurance is endogenous and the structural equation is correctly specified, we can use the IV 2SLS estimates for inference.

The linear projection of health insurance on the exogenous covariates and the vector of instruments indicates that age positively and significantly predicts health insurance status (Tables 3 and 4, column 3). Older individuals are more likely to have health insurance. Also, a primary, secondary or higher education compared to none significantly promotes health insurance uptake. Media exposure, better access to drinking water and cleaner sources of energy for cooking also promote health insurance uptake. Improvements in income from the poorest to the richest income quintile positively and significantly predicts insurance uptake. Living in a household cluster with higher proportion of insured individuals increases the likelihood of having health insurance. Those experiencing OOP were likely to have insurance coverage. Easy access to basic community level services such as nearest secondary school, road and bus stage also positively predicts health insurance uptake. Being in a single marital status, whether by choice or circumstances negatively predicts health insurance uptake. Similarly, employment in the informal sector, being unemployed and non-Christian negatively predicts health insurance uptake.

Recall that due to the endogeneity of health insurance status, the OLS parameter estimates are biased. The IV 2SLS produces consistent estimates of health status production (mortality and chronic illness). The test on the coefficients of the control variables in the CFA equation are mixed. In both models, the F-test (3.50 and 2.92) indicates that the coefficient on the residual of health insurance status is significant at *p < 0.1*, indicating a marginal possibility of heteroskedastic errors. However, the F-test (0.73 and 0.53) on the coefficient of the interaction term between health insurance status and its residual is not significant, and is an indication that any neglected non-linear interactions have been controlled for. This finding requires that we interpret the CFA findings with caution, due to possible heterogeneity of the disturbances.

Tables 3 and 4 column 2 present the OLS estimates, column 4 the IV 2SLS and column 5 the CFA estimates of the impact of health insurance on mortality and chronic illness respectively. Table 3 indicates that the OLS coefficient on health insurance status is small, positive, not statistically significant and likely to be biased. The IV 2SLS coefficient is negative and significant and is nearly five and three times larger than the OLS and CFA coefficients. The CFA coefficient, though not significant is consistent with the IV 2SLS in terms of direction of impact. The size of the IV 2SLS and CFA coefficient indicates that we are likely to underestimate the impact of health insurance on health status if we do not take endogeneity into account. We conclude that having health insurance reduces mortality. Health insurance coverage should therefore be enhanced and promoted as it confers positive health benefits to the population. The coefficients on IV 2SLS and CFA for individuals who experienced OOP expenditures indicate worse off health status. Increasing OOP expenditures leads to higher mortality, suggesting the need for mitigating measures to reduce OOP expenditures. The impact of income on health status is negative for individuals in the richest quintile for the IV 2SLS and CFA. This is an indication that improvements in incomes are associated with better health outcomes. However, for the lower income quintiles the health status impact is adverse. Marital status has positive and significant impact on health status and the size of the coefficient is almost the same in all three models. Those in special single marital status are highly and significantly likely to experience mortality. This is consistent with the finding that this category of individuals was unlikely to have health insurance. It also suggests that this may be a marginalized population group in terms of economic empowerment and subsequent health status compared to the married population.

Table 4 indicates that the IV 2SLS coefficient on health insurance is 1.2 times larger than the CFA coefficient and nearly five times larger than the OLS one. Both the IV 2SLS and CFA coefficient on health insurance status are positive and significant, contrary to the finding using mortality as the health status measure. The positive coefficient suggests that having health insurance is linked to chronic illnesses, indicating a certain degree of adverse selection. It also points to the fact that the impact of health insurance on health outcome may be sensitive to the health outcome measure used. The coefficient on age is positive, significant and similar in IV 2SLS and CFA and slightly higher in OLS models. This indicates that as people get older, chronic illnesses increase and this is consistent with both theory and empirical literature. The impact of income on chronic illness is positive and significant for all quintiles except the middle-income quintile suggesting that, the poor and the rich experience poor health as measured by the presence of chronic illnesses. Experiencing higher OOP expenditures was linked to being in a worse off health status. Being employed in the informal sector or being unemployed has a positive coefficient in all models, implying worse off health status. Increasing levels of education are associated with better health status in the IV 2SLS and CFA models. Results also indicate that those in single marital status have worse off health status in terms of chronic illnesses compared to the married.

## Discussion

An understanding of the differences between insured and uninsured populations and the impact of health insurance on health outcomes is vital for policy formulation on the value of initiatives to expand health insurance coverage. However, this is a largely under-investigated aspect in Kenya. To our knowledge, this is the first article that has utilized nationally representative data to understand the causal links between health insurance and health outcomes in Kenya. Furthermore, the estimation approach adopted accounts for potential reverse causality and heterogeneity biases. Our findings indicate that less than one-fifth of individuals were covered by any health insurance and private health insurance coverage is particularly low. This is consistent with Acharya et al.’s [31] observation that low enrollment persists for many insurance schemes in LMICs. Low insurance coverage is not conducive for financial risk protection and achievement of UHC. In addition, the extent of coverage in terms of the comprehensiveness of the insurance is important. Indeed, nearly one-quarter of individuals experienced OOP of a mean magnitude of 11% of per capita GDP (USD1410) in 2016 [83], clearly a significant proportion. In addition, the insured had higher chronic illnesses and OOP.

Both mortality and chronic illness experience were less than 4%, although nearly a quarter experienced any illness. This suggests that majority had good health status. The insured experienced lower mortality but higher illness and chronic illness suggesting the presence of adverse selection. That a higher proportion of the insured experienced OOP expenditures, though contrary to theory and arguments for health insurance, has several implications. It is plausible that ease of access to care for the insured results in higher OOP given the insurance package type and extent of co-payment. Barasa et al. [8] and Chuma et al. [84] highlight the problems with fragmentation of schemes and the adverse effects that service providers create through discriminating clients from different NHIF schemes, thereby occasioning OOP costs. It is imperative to note that the NHIF benefit packages for different population groups have exclusions and do not fully cover the healthcare costs at the point of use and for the full continuum of care even for disease conditions covered, e.g. kidney failure^2^. Ataguba and Goudge [32] observe that insurance schemes with high co-payments due to low-cost minimum-benefits choices may lead to high OOP payments and create financial burden. Secondly, the insured may also use more health services [14] in the face of adverse selection and hence incur higher OOP costs. Third, is the possibility that the existing health insurance benefit packages are inadequate requiring the insured to incur OOP expenditures. These raise the question of whether health insurance in Kenya adequately shields against OOP costs and hence financial risk protection, requiring further empirical investigation. In their systematic review, Acharya et al. [31] document that few insurance schemes afford protection from OOP spending and Kenya appears to be one of those that do not. This calls for health insurance benefit packages that are comprehensive and adequate to reduce OOP expenditures. As expected, significantly more of those in formal employment and with higher incomes are insured. Insurance uptake differed significantly across all the socio-economic and demographic characteristics. The insured are older compared to the younger population, pointing to an area that should be targeted when trying to achieve UHC. In general, more of the insured were male, living in smaller households, urban dwellers, married, Christians, with secondary and higher education and exposure to media. Improving the levels of education and limiting family sizes should be key areas of focus for influencing the socio-economic and demographic characteristics associated with higher likelihood of health insurance uptake in Kenya.

We established that there is a bi-directional causality between health insurance status and health outcomes, namely mortality and chronic illness. Though our instruments as a set were weakly relevant, they were valid and strong and therefore reliable for inference. The finding that health insurance reduces mortality is valuable for Kenya, in its quest to improve population health and the achievement of UHC goals. Health insurance coverage should therefore be enhanced and promoted as it confers positive health benefits to the population. Our finding support Stone et al. [14] in Kenya, though their study was hospital-based. Other studies with similar findings include Rand [17], Currie and Gruber [35, 36] and Culyer and Newhouse [13], though in developed countries settings. Though our findings are Kenyan specific, they can be generalized to other LMICs with similar characteristics, albeit with caution. Using chronic illness as a health outcome measure resulted in unexpected impact of health insurance on health outcomes. The finding suggests that having health insurance is linked to chronic illnesses reinforcing the observation of a certain degree of adverse selection. It also points to the fact that the impact of health insurance on health status may be sensitive to the health status measure used. This calls for primary research using valid and reliable health status measurement and valuation tools rather than relying on limited secondary data in assessing the impact of health insurance and other health interventions on health outcomes.

The finding that the young and special-single were mostly uninsured and experienced higher mortality indicates key marginalized sections of the population requiring targeting for health insurance coverage in order to improve their health status. Conversely, though the older population had insurance, chronic illness experience increased with age, consistent with both theory and empirical literature. Since, health insurance reduces mortality and chronic illnesses mostly affect the older people, we suggest that the government should establish and strengthen comprehensive health insurance programs for the elderly. This is in light of age cut-offs for most private health insurers that leave this population in need and vulnerable. Individuals from larger households had worse health status, an indication that policies to reduce household sizes may indirectly contribute to UHC and other health system goals.

We have demonstrated that though health insurance coverage is low in Kenya, it is beneficial in reducing mortality. In view of the fragmented nature of the health insurance landscape, the government should in the short-term institute policy to consolidate different health insurance schemes in order to serve different population groups more effectively and equitably. To increase coverage, NHIF should design comprehensive benefit packages within the consolidated scheme with differentiated premiums, allowing individuals to choose the benefit package and premiums within the voluntarily component, while retaining the tax funded and social security health insurance component to cushion the poor and vulnerable. This brings in the question of the informal sector who are largely without insurance. The informal sector is widely heterogeneous and it is possible that sections of it may be characterized by higher income levels. The Chilean health insurance system allows those who can afford to opt out of the publicly funded health insurance to purchase private insurance [85] with requirement for everyone to have some form of insurance. This option can be explored in Kenya in the long-term as the economy grows to allow those in the formal and informal sectors of the economy, the choice of public or private scheme, but with mandatory requirements for all to have coverage. Such a policy could work if NHIF comes up with attractive schemes for the able informal sector segment. Indeed, there is a growing trend of like-minded people coming together as groups to negotiate for attractive group cover with NHIF (e.g. retirees and community-based organizations) pointing to opportunities for and an unmet demand for health insurance.

The fact that a higher proportion of the insured experienced OOP expenditures raises concern about the adequacy of the current health insurance arrangements in mitigating catastrophic health expenditures and impoverishment. This calls for a thorough re-examination and research on the adequacy and impact of health insurance on OOP expenditures. There is need to build a further evidence base to support the argument that health insurance protects against financial risks and impoverishment in Kenya, through government support.

This study had a number of limitations. Data limitations hampered the empirical assessments of the impact of health insurance on health status. Firstly, the dominance of NHIF, low levels of private insurance and nature of health insurance whereby individuals can have more than one type of insurance constrained the analysis of impact by insurance type. Further, health insurance data collected in routine surveys (e.g., KIHBS) are not disaggregated by type of enrolment (voluntary, mandatory), contributions (risk-rated, community-rated, income-based) and management (for-profit, non-profit, public). Secondly, other than mortality measures, there is a paucity of data on health measurement and valuation in Kenya. Though valid and reliable health valuation and measurement questionnaires have not been developed for Kenya, an EQ-5D version for Kenya is available. This study recommends that in order to inform the current efforts towards UHC through health insurance, the government should institute routine measurement and valuation of population health using the available health measurement and valuation instruments. In addition, during the national surveys, data on health insurance should be disaggregated. Moreover, an immediate policy should entail routine reporting of such data to a centralized health insurance agency in coordination with KNBS to facilitate empirical studies on the benefits of various health production inputs. Thirdly, the ideal analytical techniques to infer impact would be to use either experiments, panel or time series data that would allow for lags in treatments and effects. The cross-sectional nature of the data used in this study poses a limitation in impact assessment. Future studies should explore the generation and use of such data.

## Conclusions and policy recommendations

Less than one-fifth of Kenyans are covered by any health insurance and private health insurance coverage is particularly low. The insured experienced lower mortality, more chronic illnesses and higher OOP expenditures. Experience of higher OOP expenditures may imply ease of access to care and more use of healthcare services for the insured while indicating shortcomings given the insurance package type and extent of co-payment, resulting in a financial burden. Kenya thus needs to design health insurance benefit packages that are comprehensive and adequate in coverage to reduce OOP expenditures, more so for the elderly who suffer more chronic illnesses. Targeting the young, special single and the vulnerable who were mostly uninsured and experienced higher mortality would be a desirable feature in the design of the insurance model, given the positive benefits of health insurance on health outcomes. Our finding that health insurance reduces mortality is valuable for Kenya in its quest to improve population health and the achievement of UHC goals.

We make the following policy recommendations to increase health insurance coverage as it confers positive health benefits to the population. In view of the fragmented nature of health insurance landscape, the government should in the short-term institute policy to consolidate different health insurance schemes in order to serve different population groups more effectively and equitably. To increase coverage, NHIF should design comprehensive benefit packages within the consolidated scheme with differentiated premiums, allowing individuals to choose the benefit package and premiums within the voluntary NHIF component, while retaining the tax funded and social security health insurance component to cushion the poor and vulnerable populations.

Policy reforms should also be instituted to reduce OOP and address the inadequacy of health insurance in ensuring access and protecting against financial risks. This calls for health insurance benefit packages that are comprehensive, adequate and accessible. This can be achieved through health systems strengthening to improve access to health services, institutional and governance reforms at the NHIF (the government’s preferred vehicle in the achievement of UHC) to improve and enhance benefits.

Recognizing the heterogeneous nature of the informal sector and that sections of it may be characterized by high income levels, a long-term policy recommendation is to allow those in the formal and informal sectors the choice to enroll in a public or private scheme, but with mandatory requirements for every citizen to have insurance coverage. The government should however maintain programs targeted at the poor and vulnerable populations.

## Data Availability

This research uses national survey data that is publicly available for research collected by Kenya National Bureau of Statistics. The 2015/16 Kenya Integrated Household Budget Survey (KIHBS) dataset can be accessed at (https://www.knbs.or.ke) upon reasonable request.

## Abbreviations

CFA: Control Function Approach
CSS: Civil Service Scheme
EPI: Expanded Programs on Immunization
EQ-5D: EuroQol-5 Dimension
GDP: Gross Domestic Product
GOK: Government of Kenya
HIE: Health Insurance Experiment
HISP: Health Insurance Subsidy for the Poor
IV 2SLS: Instrumental Variable 2-stage Least Squares
IV: Instrumental Variables
KES: Kenya Shillings
KIHBS: Kenya Integrated Household Budget Survey
KNBS: Kenya National Bureau of Statistics
LMICs: Low- and Middle-Income Countries
MLE: Maximum Likelihood Estimation
MoH: Ministry of Health
NASSEP V: National Sample Survey and Evaluation Programme-Five
NHIF: National Hospital Insurance Fund
NSHIF: National Social Health Insurance Fund
OLS: Ordinary Least Squares
OOP: Out-of-Pocket
RCT: Randomized Control triaT
SDG3: Sustainable Development Goal number Three
SSA: Sub-Saharan African
UHC: Universal Health Coverage
UN: United Nations
USA: United States of America
USD: United States Dollars
WHO: World Health Organization

## Footnotes

1 The average exchange rate is 1USD = KES100.2248 as at February 2019.

2 This is an example of a situation whereby NHIF covers for dialysis and kidney transplant, but does not cover the post-transplant medications which are quite expensive. This may therefore raise the OOP for patients opting for kidney transplant and dialysis.
